# Recovery of Lanthanum(III) and Nickel(II) Ions from Acidic Solutions by the Highly Effective Ion Exchanger

**DOI:** 10.3390/molecules25163718

**Published:** 2020-08-14

**Authors:** Dorota Kołodyńska, Dominika Fila, Zbigniew Hubicki

**Affiliations:** Department of Inorganic Chemistry, Institute of Chemical Sciences, Faculty of Chemistry, Maria Curie-Skłodowska University, Maria Curie-Skłodowska Sq. 2, 20-031 Lublin, Poland; dominika.fila@poczta.umcs.lublin.pl (D.F.); zbigniew.hubicki@poczta.umcs.lublin.pl (Z.H.)

**Keywords:** Lewatit Monoplus SP112, lanthanum, nickel, sorption, desorption

## Abstract

The recovery of La(III) and Ni(II) ions by a macroporous cation exchanger in sodium form (Lewatit Monoplus SP112) has been studied in batch experiments under varying HNO_3_ concentrations (0.2–2.0 mol/dm^3^), La(III) and Ni(II) concentrations (25–200 mg/dm^3^), phase contact time (1–360 min), temperature (293–333 K), and resin mass (0.1–0.5 g). The experimental data revealed that the sorption process was dependent on all parameters used. The maximum sorption capacities were found at CHNO_3_ = 0.2 mol/dm^3^, m = 0.1 g, and T = 333 K. The kinetic data indicate that the sorption followed the pseudo-second order and film diffusion models. The sorption equilibrium time was reached at approximately 30 and 60 min for La(III) and Ni(II) ions, respectively. The equilibrium isotherm data were best fitted with the Langmuir model. The maximum monolayer capacities of Lewatit Monoplus SP112 were equal to 95.34 and 60.81 mg/g for La(III) and Ni(II) ions, respectively. The thermodynamic parameters showed that the sorption process was endothermic and spontaneous. Moreover, dynamic experiments were performed using the columns set. The resin regeneration was made using HCl and HNO_3_ solutions, and the desorption results exhibited effective regeneration. The ATR/FT-IR and XPS spectroscopy results indicated that the La(III) and Ni(II) ions were coordinated with the sulfonate groups.

## 1. Introduction

Nowadays the world is dependent on natural resources mined from the earth, but unfortunately they may run out. That is also why other rare and valuable metals secondary sources are increasingly sought. There are millions of old and disused electronic and electrical devices around the world, so-called Waste Electrical & Electronic Equipment (WEEE) at our homes, i.e., old cell phones, smartphones, laptops, computers, non-working printers, and many others. At present, WEEE is becoming the fastest growing waste stream in the world [1,2,3]. In 2016 the total e-waste was 44.7 Mt worldwide, and it is expected to grow to 52.2 Mt in 2021 [4]. Each of these devices contains a wide range of valuable metals as well as hazardous substances. Improper WEEE waste management can cause serious health and environmental problems as well as loss of many precious metals. Moreover, the electronics production requires the use of limited and expensive resources. For example, waste battery materials contain many metals, for instance cobalt, lithium, nickel, and rare earths. The valued metals recovery from spent batteries not only contributes to environment protection, but also improves the resources utilization and reduces battery production costs [5]. Currently, in many areas of life, the most commonly used batteries are nickel-metal hydride batteries and lithium-ion batteries [6,7,8,9,10].

Nickel-metal hydride batteries (Ni-MH) are one of the most important components in the electronic and electric equipment. The Ni-MH batteries found applications in electric vehicles and portable electronic devices such as hybrid cars, cameras, laptops, and notebooks. These batteries are rich in nickel, cobalt, rare earth elements (i.e., lanthanum, cerium, praseodymium and neodymium), and iron [11]. Until now, the most economically essential metals applied in the nickel-metal hydride batteries production have been nickel and cobalt, but in the last decade rare earth elements gained importance as a significant material for the battery alloys production due to their increasing consumption. Therefore, due to the content of so many economically and technologically important metals in Ni-MH batteries, recycling is a very important issue. The properly recycled batteries may contribute to obtaining possibly cheap metals sources. Therefore, appropriate recycling methods are constantly being developed [6,10,11,12,13].

Metal ions recovery from different waste types most often involves the combination of pyrometallurgical and hydrometallurgical processes. For hydrometallurgical technology, after raw wastes dissolution with suitable leaching solutions, metals of specific concern should be separated from other metals. To this end ion exchange, solvent extraction, combined ion exchange/solvent extraction or liquid membrane processes can be applied [14,15,16,17]. In recent years many commercially available ion exchangers such as Amberlyst 15 [18], Amberlite IRC-748 [19], DOWEX C-500 [20], Dowex 50 × 8 [21], Purolite C-100 [22], or KU-2–8 [23] were used for heavy metal ions and rare earths removal from various types of wastes. For example Xu et al. [24] studied Co, Nd and Dy separations from the spent NdFeB permanent magnets using the am-ZrP/PAN composite ion exchanger. From their column studies, fractions with purity of 96.4% Nd, 87.9% Co, and 40% Dy were obtained. Virolainen et al. [25] investigated the recovery of rare earth elements from phosphogypsum using various types of ion exchangers, i.e., the chelating ion exchanger Purolite S940 containing the aminophosphonic functional groups and the strongly acidic cation exchangers Purolite C150 and Finex CS16GC with the sulfonic functional groups. The studies revealed that the efficiency of REE recovery in a one-step process was greater when a chelating ion exchanger was used. The application of ion exchange process in the rare earth elements sorption and separation creates the possibility of obtaining elements with a high purity degree. That is why effective ion exchangers are increasingly being sought.

In this study, two selected metal ions were used as the main metals found after leaching of spent nickel-metal hydride batteries. Our previous research proved that in such solutions the La(III) and Ni(II) concentrations are 0.7 and 2.7 g/dm^3^, respectively [26]. Therefore, the main goals of this paper were: (a) evaluation of La(III) and Ni(II) ions sorption efficiency by the macroporous ion exchanger with sulfonic functionalities under different conditions by static and dynamic experiments, (b) determination of kinetic, equilibrium, and thermodynamic parameters in order to explain the mechanism governing metal ions sorption, (c) ion exchanger characterization before and after metal ions sorption, and (d) preliminary multi-metal adsorption from binary solutions.

## 2. Results and Discussion

### 2.1. Physicochemical Characterization of Lewatit Monoplus SP112

**Figure 1:**
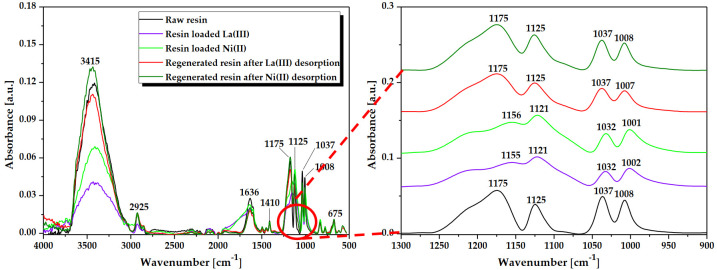
ATR/FT-IR spectra of Lewatit Monoplus SP112 before and after loading with La(III) and Ni(II) ions as well as after regeneration.

Fourier transform infrared spectroscopy with attenuated total reflectance (ATR/FT-IR) was used to recognize and verify the functional groups presence in the ion exchanger structure and also to obtain knowledge about possible interactions between the functional groups and metal ions during the sorption process. Figure 1 shows the ATR/FT-IR spectra of raw Lewatit Monoplus SP112, the La(III) and Ni(II) loaded ion exchanger as well as the regenerated ion exchanger.

As presented in Figure 1, the sharp peak observed at 3415 cm^−1^ indicates stretching vibrations of hydroxyl groups (-OH). The peak at 2925 cm^−1^ proves the presence of stretching vibrations of aliphatic groups (>CH_2_). The presence of C=C group in the benzene ring was confirmed by the peaks at 1636 and 1601 cm^−1^. Additionally, the peak appearing at 1410 cm^−1^ indicates the C-C stretching vibrations of ring. The bands in the range of 900–650 cm^−1^ are associated with the C-H aromatic out-of-plane deformation [27]. The four peaks at 1175, 1125, 1037, and 1008 cm^−1^ observed on the Lewatit Monoplus SP112 before loading are attributed to the presence of sulfonic groups. These peaks prove the presence of stretching vibrations of S=O and S-O groups in the -SO_3_Na group [28]. It can be noticed that these peaks moved away or their intensity changed after the La(III) and Ni(II) ions sorption process. After the La(III) and Ni(II) loading, the characteristic peaks for the sulfonic groups at 1175, 1125, 1037, and 1008 cm^−1^ were moved to 1155, 1121, 1032, and 1002 cm^−1^ for La(III) ions as well as to 1156, 1121, 1032, and 1001 cm^−1^ for Ni(II) ions. The most notable change was observed for the peak at 1175 cm^−1^, which after sorption was significantly reduced. These changes may be due to the bond of La(III) or Ni(II) ions with the sulfonic groups, which changed the original energy of the sulfonic groups. Thus the sorption process may be associated with the exchange of Na ions present in the SO_3_Na group for La(III) and Ni(II) ions and also the new bonds formation between the S-O, S=O groups and metal ions [29,30,31,32]. After the desorption process of La(III) and Ni(II) ions, it was found that the peaks in the spectra were recorded at the same wavenumbers as for Lewatit Monoplus SP112 before sorption, confirming the sulfonic groups restoration and the non-destructive character of the desorption process. This ATR/FT-IR observation affirms that the regenerative method is greatly efficient.

**Figure 2:**
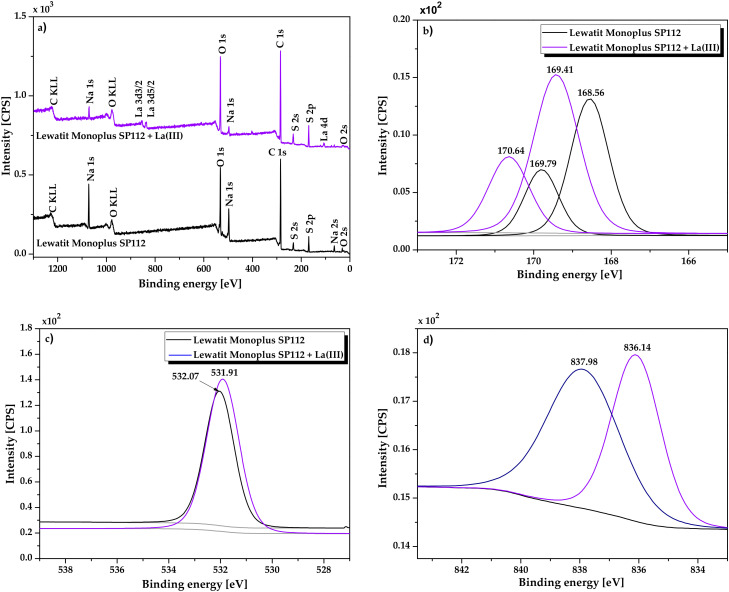
XPS spectra of Lewatit Monoplus SP112 before and after La(III) loading: (**a**) survey, (**b**) S 2p, (**c**) O 1s, and (**d**) La 3d.

X-ray photoelectron spectroscopy (XPS) was used to find out the interaction character of La(III) ions with the Lewatit Monoplus SP112 and to identify the surface functional groups present in the ion exchanger, which will allow to investigate the mechanism of La(III) ions sorption. Figure 2 presents the XPS spectra of Lewatit Monoplus SP112 before and after La(III) loading.

In the presented spectra, the bands corresponding to C 1s, O 1s, S 2p and Na 1s levels were distinguished, which confirms the basic elemental composition in the ion exchanger structure. These bands were found at the binding energies equal 285.0 eV for C 1s, 532 eV for O 1s 169.0 eV for S 2p, and 1072.5 eV for Na 1s. The atomic weight % of C, O, S, and Na before adsorption was 66.9, 20.5, 8.6, and 4.0% which changed after adsorption to 66.0, 21.9, 10.3, and 1.1%, respectively. Moreover, a new peak corresponding to La 3d in the range of 834–841.5 eV appeared after La(III) loading (0.7 atomic weight %). The carbon main forms are C=C, C-C, C-H, C-S bonds while the basic oxygen forms are proved by the S=O, S-O-Na bonds. In the S 2p spectra, the binding energy in the range of 168–171 eV correspond to S atoms in the sulfonate groups (C-SO_3_-Na) [33,34]. After La(III) ions interactions with the Lewatit Monoplus SP112 structure, the binding energies of the S 2p level shifted towards higher values (from 168.56 and 169.79 to 169.41 and 170.64, respectively). Additionally, in the O 1s spectrum the peak at 532.07 eV is assigned to O (-SO_3_) in the sulfonate groups. After adsorption, this peak was shifted to 531.91 eV, indicating new La-O bonds formation. It was also noticed that after La(III) ions sorption, the peaks corresponding to Na 1s level were significantly reduced. The Na ions content in the Lewatit Monoplus SP112 structure was reduced from 4.0 to 1.1% while the La(III) ions content increased from 0.0 to 0.7%. The changes may indicate ion exchange during the sorption process and also the participation of sulfonate groups in the coordination of lanthanum(III) ions.

**Figure 3:**
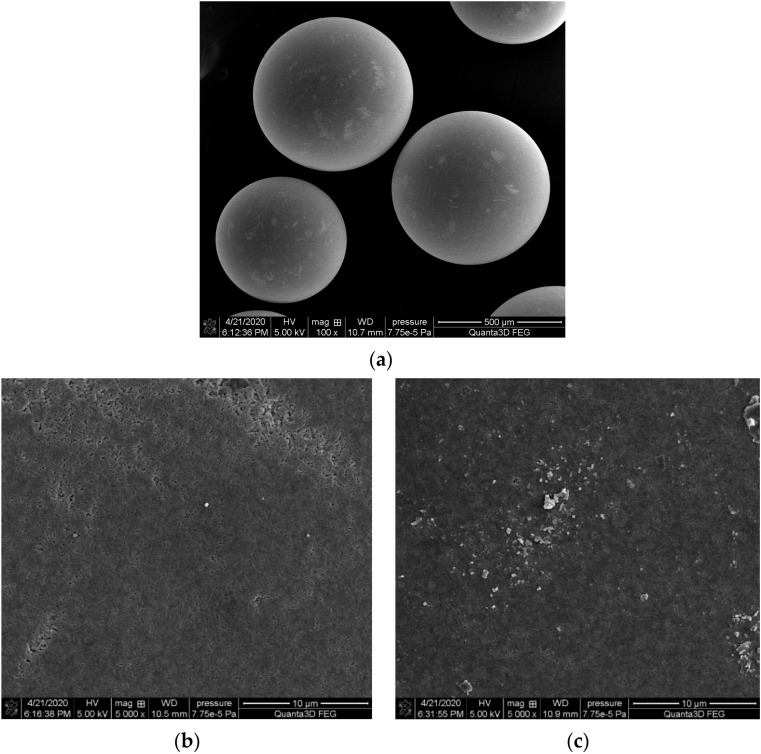
SEM micrographs of Lewatit Monoplus SP112 (**a**) spherical shape of ion exchanger, (**b**) before and (**c**) after La(III) loading.

The use of scanning electron microscopy makes it possible to learn the morphology and the topography of ion exchanger as well as its porosity, which is necessary to assess adsorption abilities. For this purpose, SEM images were recorded using a 3D FEG scanning electron microscope (Quanta, Hillsboro, OR, USA). Figure 3 shows the SEM images of ion exchangers before and after the La(III) ions sorption at 100× and 5000× magnifications.

As shown in Figure 3a, the ion exchanger beads have a spherical shape, but differ in size, indicating that the ion exchanger is polydispersive. It was also observed that the Lewatit Monoplus SP112 surface is rather homogeneous and porous which may confirm the presence of pores in the ion exchanger structure (Figure 3b). These pores are a suitable place for the sorption of metal ions. After the La(III) ions sorption process (Figure 3c), changes in the SEM images were noticed, which could confirm the adsorption of La(III) ions onto the Lewatit Monoplus SP112 surface. Characterization of the porous structure parameters of the ion exchanger, i.e., the specific surface area, pore size and pore volume was determined using the low-temperature nitrogen adsorption/desorption isotherms obtained on an ASAP 2405 sorption analyzer (Micromeritics, Norcross, GA, USA).

Table 1 summarizes the values of obtained parameters.

**Figure 4:**
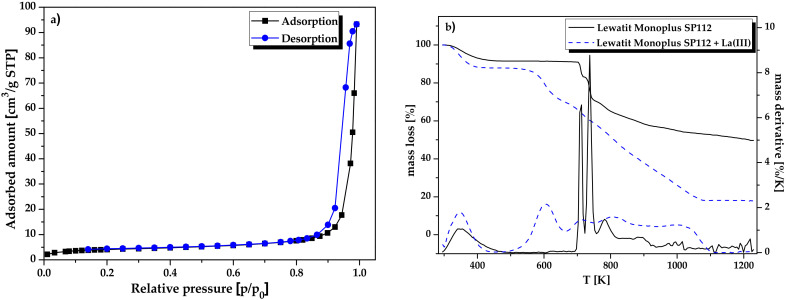
(**a**) Low-temperature adsorption/desorption nitrogen isotherm and (**b**) TG/DTG curves of Lewatit Monoplus SP112.

The specific surface area was not very large, because it assumes a small value of 14.98 m^2^/g. The total pore volume was 0.144 cm^3^/g while the mean pore size was equal to 32.72 nm. The obtained pore size values confirm the presence of mesopores (range 2–50 nm) in the ion exchange structure. Figure 4a shows the low-temperature nitrogen adsorption and desorption isotherms for Lewatit Monoplus SP112. According to the IUPAC classification, the shape of low-temperature nitrogen adsorption/desorption isotherms is characteristic of type II with a hysteresis loop at a relative pressure of about 0.9. Type II is characteristic of non-porous or macroporous materials. In addition, according to the IUPAC classification, the obtained hysteresis loops belong to the H3 type [35]. This type is characteristic of mesoporous materials composed of agglomerated pores with a wide size distribution and indicates the open pores structure [36].

A thermal analysis method was also used to assess thermal stabilities of samples before and after La(III) ions sorption. Figure 4b presents the TG and DTG curves for Lewatit Monoplus SP112 before and after La(III) loading. Based on the curves course, it was found that their decomposition takes place in several steps. In the first step, when the temperature increased from 295 K to 434 K, the mass loss was about 8.08% which can be ascribed to the evaporation of surface and mesopore bound water [37]. Lewatit Monoplus SP112 is characterized by high thermal stability. The decomposition of sulfonic functional groups followed by the polystyrene-divinylbenzene matrix proceeded at a temperature above 700 K [28]. For Lewatit Monoplus SP112, the total mass loss was 50.35% as the temperature increased to 1230 K. Comparing the TG and DTG curves before and after the La(III) ions sorption, it was noticed that the thermal stability after La(III) loading was reduced. The thermal decomposition of sulfonic groups began already at 564 K. Moreover, the total mass loss was about 82.13% when the temperature increased to 1230 K.

Finally, the point of zero charge measurements were conducted using the potentiometric titration method. It is known that if pH <pH_pzc_, cations sorption is favourable (Equation (1)) whereas if pH >pH_pzc_, anions sorption takes place (Equation (2)):

*R–SO_3_H + H_3_O^+^ → R–SO_3_H_2_^+^ + H_2_O* (1)

*R–SO_3_H + OH^–^ → R–SO^–^ + H_2_O* (2)

where *R* is the Lewatit Monoplus SP112 matrix.

As presented in Table 1 the obtained pH_pzc_ value was about 6.61. In this study, the optimum pH was selected as 1.5. Therefore, under these conditions the sorption effectiveness should not be large. However, %S values for the La(III) and Ni(II) ions sorption on Lewatit Monoplus SP112 were equal to 99.05% and 82.85%, respectively. This indicates that the surface charge of the ion exchanger is not so important during the sorption process. In this case chemical affinity of the ion exchanger for the adsorbed metal ions may be more crucial.

### 2.2. Basic Parameters Effecting on Batch Sorption Experiments

#### 2.2.1. Effect of HNO_3_ Concentration and Ion Exchanger Mass on Lanthanum(III) and Nickel(II) Sorption

**Figure 5:**
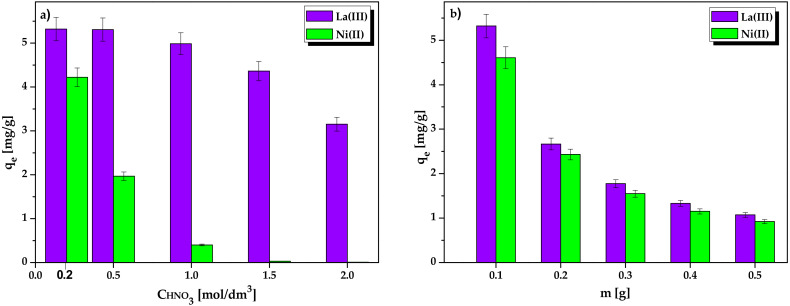
Effect of (**a**) HNO_3_ concentration and (**b**) ion exchanger mass on the La(III) and Ni(II) ions sorption by Lewatit Monoplus SP112.

The influence of the HNO_3_ concentration on the metal sorption was investigated in solutions in the range of 0.2–2.0 mol/dm^3^ HNO_3_. As presented in Figure 5a, Lewatit Monoplus SP112 shows the greatest preference for the La(III) and Ni(II) ions at 0.2 mol/dm^3^ HNO_3_, so this concentration was used in further experimental studies. The ion exchanger shows particularly strong preferences for La(III) ions in the entire tested HNO_3_ concentration range. Sorption effectiveness from the 0.2–2.0 mol/dm^3^ HNO_3_ solution decreased from 99.05% to 56.78%. For Ni(II) ions, the sorption from the 0.2–2.0 mol/dm^3^ HNO_3_ solution decreased significantly from 82.85% to 2.17%. The decrease in sorption efficiency at higher HNO_3_ concentrations (higher H^+^ ion concentration) can be explained by greater competition for the free active sites of the ion exchanger between H^+^ and La(III) or Ni(II) ions present in the solution.

Determining the optimum of the ion exchanger mass is an important stage in finding the maximum possible La(III) and Ni(II) recovery. For this purpose, a series of La(III) and Ni(II) ions solutions (10 cm^3^ each) were shaken at varying ion exchangers dosages (0.1–0.5 g). The optimum mass of Lewatit Monoplus SP112 was found to be 0.1 g with as large La(III) and Ni(II) recovery as 99.05% and 96.32%, respectively. The sorption equilibrium capacities, q_e_, were calculated to be 5.32 mg/g for La(III) and 4.61 mg/g for Ni(II) (Figure 5b). Therefore, 0.1 g was considered as the optimum mass and was used in further experiments.

#### 2.2.2. Effect of Phase Contact Time and Initial Metal Concentration on the Lanthanum(III) and Nickel(II) Sorption

**Figure 6:**
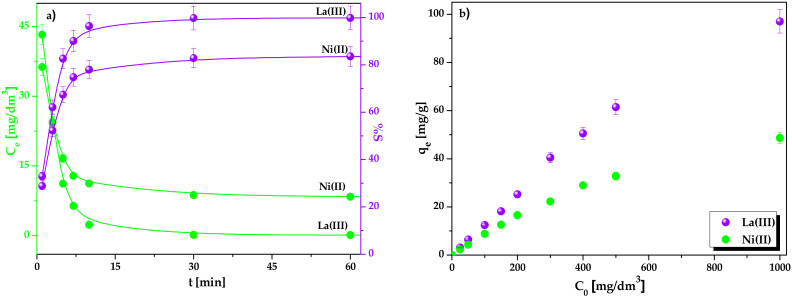
Effect of (**a**) phase contact time and (**b**) initial metal concentration on the La(III) and Ni(II) ions sorption by Lewatit Monoplus SP112.

The effect of phase contact time on the La(III) and Ni(II) ions sorption process conducted with the varied contact time from 1 to 360 min at the initial metal concentration equal 50 mg/dm^3^ at 293 K was studied. Figure 6a presents the obtained results for the La(III) and Ni(II) ions sorption onto Lewatit Monoplus SP112. Sorption efficiencies (%S) increased with the increasing phase contact time gradually. After about 3 min, 62% La(III) and 52% Ni(II) ions sorption were obtained whereas 99% La(III) and 84% Ni(II) removal took 30 and 60 min, respectively which corresponds to the equilibrium time. During the sorption process, the equilibrium metal concentration, C_e_, decreased with the increasing contact time [38]. The sorption equilibrium capacities of Lewatit Monoplus SP112 were determined to be 5.44 mg/g for La(III) and 4.31 mg/g for Ni(II) for 0.1 g resin amount and metal concentration of 50 mg/dm^3^.

Moreover, the influence of initial lanthanum(III) and nickel(II) concentrations on the sorption process conducted at the varied metal concentrations from 25 to 1000 mg/dm^3^ for 360 min at 293 K was studied. As can be seen from Figure 6b, the higher C_o_ values, the higher q_e_ values. The sorption equilibrium capacities for the initial metal concentrations in the range of 25–1000 mg/dm^3^ increased from 3.13 mg/g to 97.06 mg/g for La(III) ions and from 2.35 mg/g to 48.64 mg/g for Ni(II) ions.

### 2.3. Desorption Experiments

**Figure 7:**
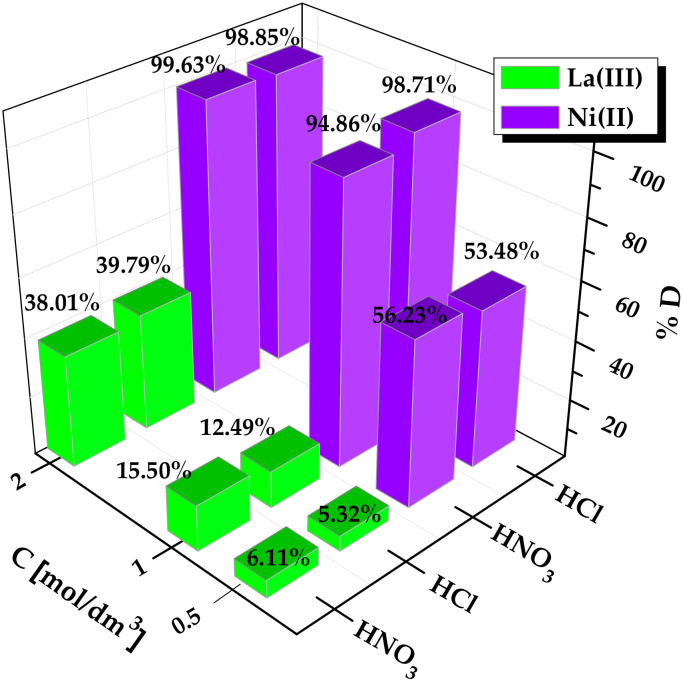
Regeneration efficiency of La(III) and Ni(II) ions on Lewatit Monoplus SP112.

Reusability abilities of ion exchangers are one of the essential factors to assess their success in commercial applications. The desorption process is required to restore the original ion exchangers adsorption capacity, and recover valuable metal ions from solutions. Desorption of La(III) and Ni(II) ions from Lewatit Monoplus SP112 was investigated using 0.5, 1, and 2 mol/dm^3^ HCl and HNO_3_ as eluents. The desorption studies were carried out under the identical conditions for La(III) and Ni(II) ions. Figure 7 shows the results obtained for individual metal ions.

When the eluents concentration increased from 0.5 to 2.0 mol/dm^3^, the desorption percentage, %D, increased. This was observed for both HNO_3_ and HCl eluents. The best results were obtained using 2 mol/dm^3^ HNO_3_ and HCl. As can be seen in Figure 7 much higher desorption efficiency was obtained for Ni(II) ions. However, desorption of La(III) ions from Lewatit Monoplus SP112 was definitely lower, maybe due to its stronger affinity for the ion exchanger. The incomplete desorption of metal ions from the surface of Lewatit Monoplus SP112 suggested the involvement of strong chemisorption mechanisms between the ion exchangers and metal ions.

### 2.4. Kinetic Parameters for Lanthanum(III) and Nickel(II) Sorption

The sorption kinetics of the La(III) and Ni(II) ions from the acidic solutions was investigated. Generally, a reaction time of 30 min for La(III) and 60 min for Ni(II) and the selected optimum conditions, i.e., 0.2 mol/dm^3^ HNO_3_ and 0.1 g of ion exchanger, were sufficient to achieve a maximum sorption yield. The obtained kinetic data of La(III) and Ni(II) were fitted to the following kinetic models: the pseudo-first and pseudo-second order as well as intraparticle diffusion, Boyd, and film diffusion models. Within the kinetic research, the film and pore diffusion coefficients were also determined. The evaluation of kinetic parameters is shown in Table 2.

**Figure 8:**
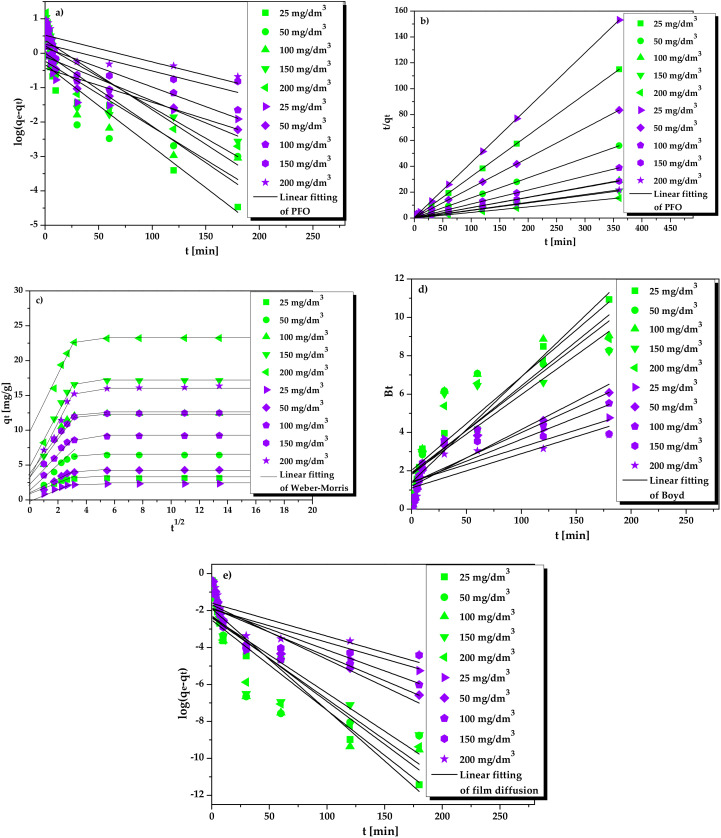
Experimental data fitting for La(III) (green symbols) and Ni(II) (violet symbols) ions sorption on Lewatit Monoplus SP112 for the (**a**) pseudo-first order, (**b**) pseudo-second order, (**c**) Weber-Morris intraparticle diffusion, (**d**) Boyd, and (**e**) film diffusion kinetic models.

At the beginning, the kinetic parameters obtained for the most commonly used pseudo-first and pseudo-second order kinetic models were compared. The plots linearity indicated the pseudo-second order kinetic mechanism of La(III) and Ni(II) ions sorption. The correlation coefficients, *R^2^*, of the pseudo-second order kinetics (0.999 for La(III) and 0.999–1.000 for Ni(II)) are greater than those of the pseudo-first order (0.711–0.949 for La(III) and 0.579–0.839 for Ni(II)). The kinetic models linear fitting is also confirmed in Figure 8a–e.

Moreover, the calculated equilibrium capacities according to the PFO and PSO kinetic models (*q_1_* and *q_2_,* respectively) were compared with the experimental equilibrium capacities, *q_exp_.* It was found that the *q_2_* values are consistent with the *q_exp_* ones. These values differ from each other by a maximum of 0.07. In contrast, the *q_1_* values were significantly lower than the *q_exp_* values. The *k_2_* rate constant values determined according to the PSO model indicate that the sorption process rate decreased with the increasing initial La(III) and Ni(II) concentrations (from 25 to 200 mg/dm^3^). The obtained results suggest that sorption may be slower at higher concentrations.

The kinetic parameters of intraparticle diffusion, Boyd, and film diffusion models as well as film and pore diffusion coefficients were calculated to determine the process governing the La(III) and Ni(II) sorption. The estimated parameters were listed in Table 2. Figure 8c–e shows the Weber-Morris intraparticle diffusion, Boyd, and film diffusion plots for La(III) and Ni(II) ions sorption by Lewatit Monoplus SP112. According to the Weber-Morris intraparticle diffusion model (Figure 8c), the La(III) and Ni(II) sorption proceeded in three steps. At the beginning the film diffusion, where metal ions were captured by the external surface sites of the ion exchanger, takes place. In the second step, the sorption process becomes slower and metal ions diffuse into the internal Lewatit Monoplus SP112 structure, known as intraparticle diffusion. The last step is a gradual sorption process, where equilibrium is established. Table 2 presents the intraparticle diffusion rate constants, *k_i1_*, *k_i2_*, and *k_i3_*, as well as C_1_, C_2_, and C_3_, which allow to estimate the boundary layer thickness. When the initial metal concentration increased from 25 to 200 mg/dm^3^ the *k_i1_*, *k_i2_*, *k_i3_*, as well as C_1_, C_2_, and C_3_ parameters also increased. The values of parameters *k_i1_*, *k_i2_*, and *k_i3_* decreased in the series *k_i1_*>*k_i2_*>*k_i3_* while the values of C_1_, C_2_, and C_3_ increased in the series C_3_> C_2_> C_1_. Large values of the intercepts of the plots indicate the effect of the boundary layer thickness on the sorption process [39]. Considering the R^2^ values for the three sorption steps, the highest values were obtained in the first step, i.e., film diffusion. In this step, for La(III) ions sorption the R^2^ values were in the range 0.965–0.984, while for Ni(II) ions sorption 0.950–0.993. Figure 8c shows that the plots are linear and do not pass through the origin. This indicate that the intraparticle diffusion process does not play a main role in the process rate controlling. These results show that the film diffusion is involved mainly in the La(III) and Ni(II) ions sorption onto Lewatit Monoplus SP112. According to the Boyd model, when the dependence of *Bt* vs. *t* is linear and passes through the origin, it means that the intraparticle diffusion is the slowest step in the sorption process. As can be seen in Figure 8d the plots do not pass through the origin of the coordinate system, confirming minor contribution of intraparticle diffusion to metal ions sorption. The *R^2^* values for the Boyd model were 0.711–0.949 for La(III) ions and 0.579–0.839 for Ni(II) ions. For the film diffusion model it was found that the film diffusion rate constants values, *k_f_*, were in the range 8.73–11.43 1/min for La(III) ions and 4.37–6.58 1/min for Ni(II) ones. Based on the above sorption kinetic studies it was found that the sorption process is mainly connected with the film diffusion and to a lesser extent with the intraparticle diffusion. To confirm the obtained results, the film and pore diffusion coefficients were also calculated (Table 2). The determined *D_f_* and D_p_ values were in the range 2.04 × 10^−7^–9.12 × 10^−6^ cm^2^/s and 8.80 × 10^−9^–1.76 × 10^−8^ cm^2^/s for La(III) as well as 1.29 × 10^−7^–6.76 × 10^−6^ cm^2^/s and 5.97 × 10^−9^–8.80 × 10^−9^ cm^2^/s for Ni(II), respectively. These results affirm that the film diffusion is the main rate controlling step of the sorption process since the *D_f_* values were in the range 10^−6^–10^−8^ cm^2^/s, as reported by Michelsen et al. [40].

### 2.5. Equilibrium Isotherm and Thermodynamic Parameters for Lanthanum(III) and Nickel(II) Sorption

**Figure 9:**
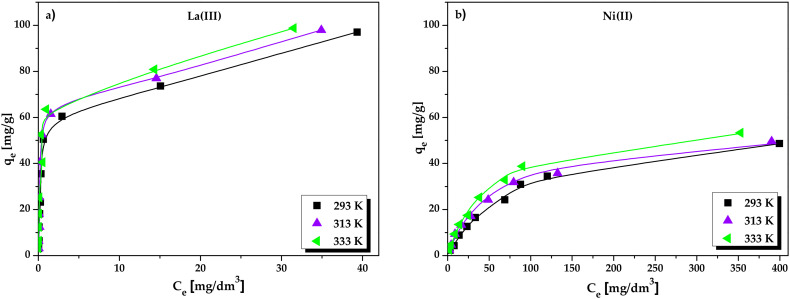

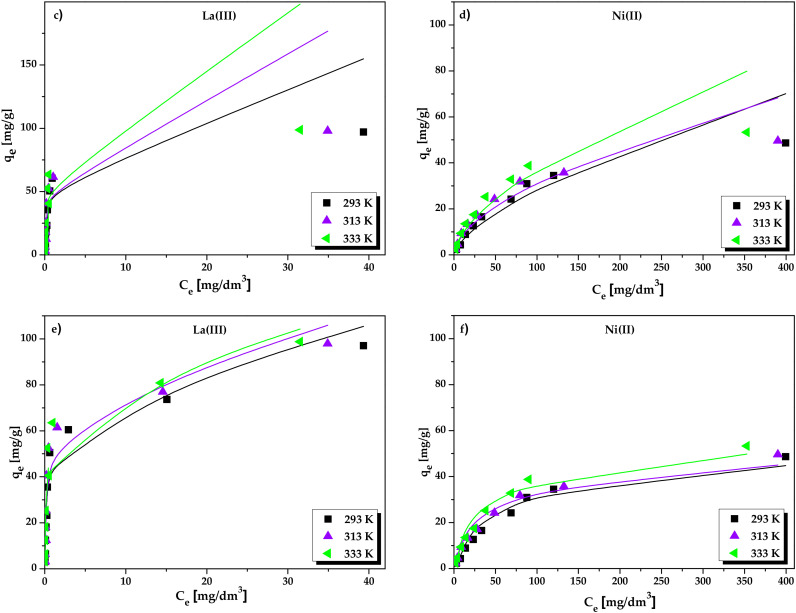
(**a**,**b**) Langmuir, (**c**,**d**) Freundlich, and (**e**,**f**) Temkin plots for the La(III) and Ni(II) ions sorption on Lewatit Monoplus SP112 at different temperatures. The symbols stand for the experimental data and the lines stand for the fitting by Langmuir, Freundlich and Temkin isotherm models.

Adsorption isotherm modelling was conducted to identify the mechanism of the La(III) and Ni(II) ions interactions with the Lewatit Monoplus SP112. The adsorption isotherms of metal ions on the ion exchanger were measured at the concentrations ranging from 25 to 1000 mg/dm^3^. The adsorption isotherms of La(III) and Ni(II) ions at different temperatures are illustrated in Figure 9.

The adsorption of La(III) and Ni(II) ions increased with the increasing initial metal ions concentrations and reached saturation at high concentrations. It was also noticed that the ion exchanger adsorption capacities increased with the temperature increase from 293 to 333 K. This indicates the endothermic nature of the metal ion sorption process. The Langmuir, Freundlich, and Temkin model parameters as well as the correlation coefficient (*R^2^*), Chi-square (*Χ^2^*), and root mean square error (RMSE) are presented in Table 3.

For the La(III) ions sorption, the theoretical maximum monolayer adsorption capacities are obtained to be 93.21, 94.61, and 95.34 mg/g at 293, 313 and 333 K, respectively. In the case of Ni(II) ions, the *q_m_* values were equal to 55.30, 59.17, and 60.81 mg/g at 293, 313 and 333 K, respectively. The calculated experimental adsorption capacities (*q_exp_*) were consistent with the *q_m_* values. The isotherm results indicate that the monolayer adsorption of La(III) and Ni(II) on the Lewatit Monoplus SP112 surface dominates the process. Additionally, the metal binding is treated as a chemical reaction between the metal ions and empty sites of ion exchanger. The Langmuir constant (*K_L_*) indicates the extent of interactions between the metal ions and the ion exchanger surface. If the *K_L_* values are relatively larger, this indicates that there are strong interactions between the metal ions and the adsorbent while smaller *K_L_* values indicate weaker interactions. As shown in Table 3, the higher *K_L_* values were obtained for La(III) ions sorption which confirms stronger interactions of La(III) ions with the Lewatit Monoplus SP112 surface. The Freundlich isotherm is not found in good agreement with the experimental data because it is applied to the adsorption on heterogeneous surfaces. The obtained values of 1/*n* below 1 indicate a normal adsorption [41]. The larger *K_F_* values for La(III) ions sorption indicate the greater adsorption ability. The B values according to the Temkin model were found to be high which indicates that the strong interactions between the metal ions and the ion exchanger. Moreover, larger values of parameter *A* corresponding to the maximum binding energy were obtained for La(III) ions, which were equal to 27.093–43.038 dm^3^/g. For Ni(II) ions, the *A* values were in the range of 0.217–0.372 dm^3^/g.

In general, when errors function values i.e., Chi-square (Χ^2^) and Root mean square error (RMSE) values are low, and the correlation coefficient (*R^2^*) values are close to 1, this indicates better agreement between the calculated and experimental data and the isotherm model gives the best fitting [42,43]. It was found that the Langmuir model provides better fitting with the experimental results than the Freundlich and Temkin models (Figure 9). The correlation coefficient for the Langmuir isotherm (*R^2^*: 0.996–0.999 for La(III) and 0.990–0.998 for Ni(II)) was higher than that for the Freundlich (*R^2^*: 0.594–0.699 for La(III) and 0.954–0.959 for Ni(II)) and Temkin (*R^2^*: 0.896–0.921 for La(III) and 0.936–0.959 for Ni(II)) isotherm model. Moreover, the lowest values of Χ^2^ and RMSE obtained for the Langmuir isotherm model confirmed satisfactory fitting to the experimental data. Based on the *R*^2^, Χ^2^, and RMSE values, the best of the adsorption system order are as follows: Langmuir > Temkin > Freundlich for La(III) and Ni(II) ions sorption process.

**Figure 10:**
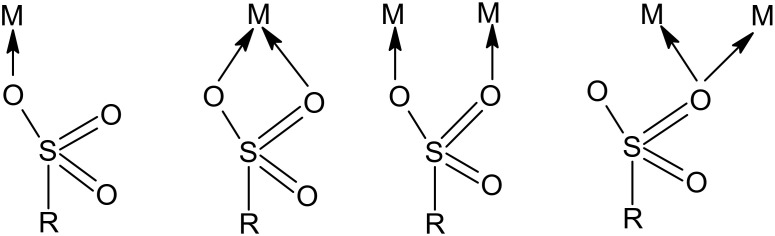
Possible metal coordination with sulfonate groups (own elaboration based on [45]).

It is well known that sulfonate groups contain O atom as a donor atom [44,45]. According to Côté and Shimizu [45] each oxygen atom in the sulfonate group can bind more than one metal ion. Oxygen atoms can most often bind maximum two metal ions. Figure 10 shows the possible metal coordination for the sulfonate group (where M is the metal and R is the matrix structure).

The obtained XPS and ATR/FT-IR results revealed that the functional groups in the Lewatit Monoplus SP112 structure are successfully involved in the La(III) and Ni(II) ions sorption process. These results confirm the participation of sulfonate groups in the coordination of metal ions and the new bond La-O and Ni-O formation. The changes in the ATR/FT-IR and XPS spectra may also indicate the exchange of Na ions into Ni(II) or La(III) ions during the sorption process.

**Figure 11:**
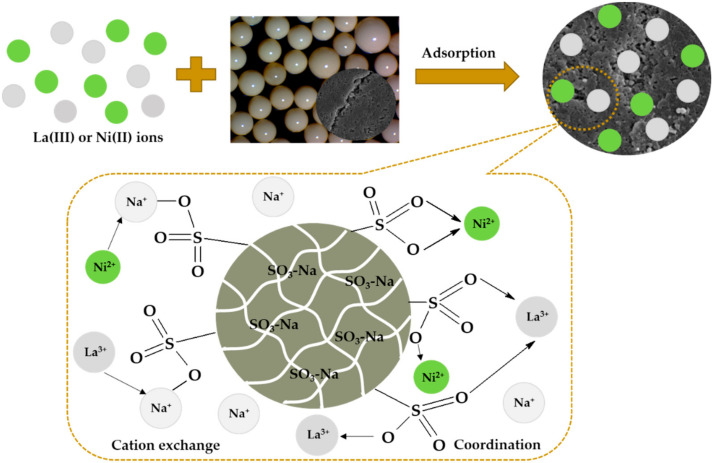
Probable La(III) and Ni(II) ions interactions with Lewatit Monoplus SP112.

A graphic representation of the metal ions sorption mechanism is shown in Figure 11.

The lanthanum(III) and nickel(II) adsorption performance were correlated to the other materials, as presented in Table 4, where the maximum adsorption capacities, equilibrium times, pH values, and temperature were compared. Among the sorbents reported in the literature, Lewatit Monoplus SP112 has much greater the *q_e_* values and the equilibrium time is the shortest, so it is a very efficient adsorbent with good kinetic characteristics.

The La(III) and Ni(II) ions sorption process was conducted at different temperatures from 293 to 333 K at pH 1.5. The sorption of La(III) and Ni(II) ions increased by about 1.79% and 8.78%, respectively as the temperature increased from 293 to 333 K which showed the endothermic nature of metal ions sorption onto Lewatit Monoplus SP112. The temperature dependence on the sorption process is connected with some thermodynamic parameters i.e., ΔH°, ΔS°, and ΔG°. The thermodynamic parameters were calculated using the van’t Hoff equation. The ΔH°, ΔS°, and ΔG° values are reported in Table 5.

The negative ΔG° values implied that the sorption process was spontaneous. It was also found that the ΔG° values decreased gradually with the increasing temperature, suggesting that the sorption process of La(III) and Ni(II) ions on Lewatit Monoplus SP112 became more favourable with the temperature increase. The positive value of ΔH° confirmed that the process has an endothermic character. Moreover, the positive ΔS° values implied that the degree of freedom increased at the solid solution interface during metal ions sorption.

### 2.6. Column Experiment Results

**Figure 12:**
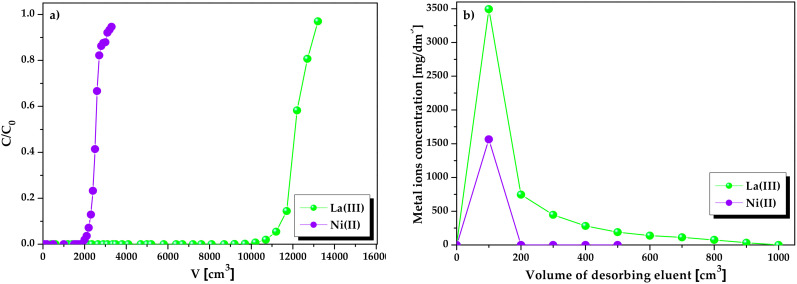
(**a**) Breakthrough curves comparison of La(III) and Ni(II) ions sorption for Lewatit Monoplus SP112 and (**b**) Elution curves for La(III) and Ni(II) regeneration.

During the column experiments, 50 mg/dm^3^ of La(III) and Ni(II) ions solution (pH = 1.5) was passed through the column packed with the 10 cm^3^ of Lewatit Monoplus SP112 at a flow rate of 0.6 cm^3^/min (Figure 12). The column studies parameters were calculated from the breakthrough curves (C/C_0_ vs. V).

The breakthrough curves shown in Figure 12a for La(III) and Ni(II) ions are consistent with the previous batch experiments. Lewatit Monoplus SP112 had a greater affinity for lanthanum(III) ions. The breakthrough data for the ion exchanger are given in Table 6. Comparing the results obtained for La(III) and Ni(II), lanthanum(III) was not present in the solution until 9200 cm^3^ of the solution was passed, while nickel(II) began to appear after about 1800 cm^3^.

After passing about 3500 cm^3^ of Ni(II) ions solution through the column, Lewatit Monoplus SP112 gets saturated. In the case of La(III), approximately 13,000 cm^3^ of La(III) ions solution was needed for the column saturation. The total and working exchange capacities as well as the volumetric and mass distribution coefficient values assumed higher values for La(III) ions than for Ni(II) ions. After the bed gets exhausted, the regeneration process to recover the adsorbed metal ions becomes quite essential. In this study, 2 mol/dm^3^ HCl solution (chosen from the static desorption studies) was found to be effective in the La(III) and Ni(II) ions recovery from the adsorption bed. The regeneration efficiency was very high being 99.96 and 99.94% for La(III) and Ni(II) ions, respectively. As illustrated in Figure 12b, elution of Ni(II) ions was easier and faster than that of La(III). Complete regeneration of Lewatit Monoplus SP112 from Ni(II) ions was achieved after passing 200 cm^3^ of 2 mol/dm^3^ HCl solution. On the other hand, complete elution of La(III) ions from the column took place after passing 1000 cm^3^ of 2 mol/dm^3^ HCl solution. It can be concluded from the column studies that Lewatit Monoplus SP112 is a high-efficiency ion exchanger, particularly for La(III) ions recovery from acidic solutions.

### 2.7. La(III) Ions Sorption Studies in the Binary System

**Figure 13:**
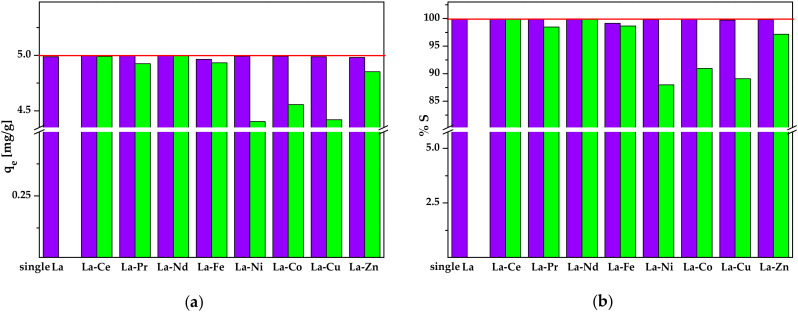
(**a**) Equilibrium sorption capacities and (**b**) Sorption percentages of La(III) ions in the different sorption systems.

There are many papers available in the literature about the metal ions adsorption by various types of adsorbents in the one-component systems. Real industrial wastewaters may contain a mixture of metal ions, so it seems necessary to study the simultaneous sorption of two or more metal ions as well as to quantify the mutual influence of one metal ion on another. As mentioned above, after nickel-metal hydride batteries leaching, the main metals in solution are nickel and lanthanum, but the other metals with a lower concentration are also present. These are cobalt, iron, zinc, copper as well as cerium, neodymium, and praseodymium. Therefore, the removal efficiency of Lewatit Monoplus SP112 for La(III) ions in the presence of the other metal ions, i.e., Ce(III), Pr(III), Nd(III), Fe(III), Ni(II), Co(II), Cu(II), and Zn(II) was estimated and the results are shown in Figure 13.

In the sorption process of a metal ion mixture, there are three possible mechanism types, i.e., synergism, antagonism and no interaction. In the first case the metal mixture effect on the sorption is greater than that of individual metal ions in the mixture. As for antagonism the metal mixture effect on sorption efficiency is smaller than that of each of the metal ions in the mixture, and in the last case the mixture does not affect the sorption of each metal ion in the mixture [51,52]. To analyse the mutual influence of the two components on the sorption process, the La(III) ions sorption efficiency in a one-component and two-component system was compared. The La(III) ions sorption efficiency in a one-component system for a concentration of 50 mg/dm^3^ was 99.85%. It is shown in Figure 13 that the presence of Ce(III), Pr(III), Nd(III), Ni(II), Co(II), Cu(II) and Zn(II) ions did not affect the sorption of La(III) ions. Only in the case of the La-Fe system a slight decrease in the efficiency of La(III) ions sorption was observed (by about 0.72%). It may be due to the competitive adsorption between La(III) and Fe(III) ions (the same oxidation state) and thus lower binding sites accessibility [53]. Lewatit Monoplus SP112 was characterized by better sorption properties for the metal ions at the +III oxidation state, i.e., La(III), Ce(III), Pr(III), Nd(III), and Fe(III) compared to the metal ions at the +II oxidation state, i.e., Ni(II), Co(II), Cu(II), and Zn(II). As commonly known, the ion exchanger affinity for the metal ions is usually associated with the chemical properties of metal ions, i.e., ionic radius, atomic mass or oxidation state. The metal ions sorption efficiency at the +III oxidation state was in the range of 98.48–99.97%. In turn, the efficiency of metal ions sorption at the +II oxidation state was 87.97–97.16%.

## 3. Conclusions

This paper investigated the sorption process of La(III) and Ni(II) ions on Lewatit Monoplus SP112 by conducting batch and column experiments under different conditions. The obtained results revealed that the maximum sorption capacities can be reached in the batch experiments under the optimum operating conditions: 0.1 g of resin dosage, HNO_3_ concentration of 0.2 mol/dm^3^, temperature 333 K, and contact time 30 min for La(III) and 60 min for Ni(II). The La(III) and Ni(II) ions sorption onto Lewatit Monoplus SP112 was well fitted by the Langmuir sorption isotherm and exhibited maximum monolayer adsorption capacities of La(III) and Ni(II) ions corresponding to 95.34 and 60.81 mg/g at 333 K. The kinetic data revealed that the La(III) and Ni(II) sorption rate was fast and well fitted by the pseudo-second order kinetic model. Moreover, the kinetic data analysis of the film and the intra-particle diffusion models revealed that the sorption process is mainly connected with the film diffusion and to a lesser extent with the intraparticle diffusion. The metal ions sorption process was endothermic, spontaneous, and favourable based on the calculated thermodynamic parameters. The adsorbed lanthanum(III) and nickel(II) ions were effectively regenerated with 2 mol/dm^3^ solutions of HNO_3_ and HCl. The column experiments show the possibility of La(III) and Ni(II) recovery from the acidic solutions and the applicability of resin regeneration. The preliminary studies of sorption from the binary solutions proved that the presence of trivalent Fe(III) ions results in aslight decrease of the La(III) sorption capacity. The presence of the other trivalent and divalent metal ions did not affect the La(III) ions sorption on Lewatit Monoplus SP112. Combined with the XPS and ATR/FT-IR results, it can be concluded that the sulfonate groups in the Lewatit Monoplus SP112 structure are successfully involved in the La(III) and Ni(II) ions sorption process.

## 4. Materials and Methods

### 4.1. Materials in Experiments

The commercial synthetic ion exchanger Lewatit Monoplus SP112 in the sodium form used in the studies was supplied by Lanxess (Cologne, Germany). The physicochemical characteristics are presented in Table 1. Prior to the experiments, the ion exchanger was washed with distilled water to remove impurities and then left for complete drying at room temperature. The prepared ion exchanger was stored in a plastic bottle for experimental research. In the experiments, the lanthanum(III) and nickel(II) ions were used to study the applicability of Lewatit Monoplus SP112 in their sorption. To this end the high purity powder of La_2_O_3_ (>99.9% purity, Merck, Darmstadt, Germany) and hexahydrate salts of Ni(NO_3_)_2_·6H_2_O (analytically pure, Avantor Performance Materials, Gliwice, Poland) were applied for solutions preparation. The standard stock solution of 1000 mg/dm^3^ La(III) was prepared by calcinating of La_2_O_3_ powder in a muffle furnace at 1073 K for 4 h. After cooling in a desiccator and weighing 1.173 g La_2_O_3_ to prepare a 1000 mg/dm^3^ solution, the oxide was digested with concentrated HNO_3_ (65% analytically pure, Chempur, Piekary Śląskie, Poland) to obtain a colourless solution. Then the standard stock solution of 1000 mg/dm^3^ Ni(II) was prepared by dissolving 4.955 g Ni(NO_3_)_2_·6H_2_O in 1 dm^3^ of 0.2 mol/dm^3^ HNO_3_ in distilled water (pH value of 0.2 mol/dm^3^ HNO_3_ solution was equal to 1.5).

### 4.2. Analytical Methods

720-ES Inductively Coupled Plasma Optical Emission Spectrometer (ICP-OES, Varian Inc.Palo Alto, CA, USA) operating with the argon gas plasma was applied to analyze the La(III) ions concentrationin solutions before and after the sorption process. The used wavelength for La(III) ions was 333.749 nm. Spectr AA240 FS Atomic Absorption Spectrophotometer (AAS, Varian Inc.) operating with an air-acetylene flame was used to analyze the concentration of Ni(II) ions. The wavelength for Ni(II) ions was 232.0 nm. The pH measurements were performed using the PHM 82 Standard pH meter (Radiometer, Copenhagen, Denmark). The laboratory shaker type 358A (Elpin+, Lubawa, Poland) at amplitude 8 and stirring rate 180 rpm was used for the batch experiments.

For the characterization of Lewatit Monoplus SP112, the Fourier transform infrared spectrometer (ATR/FT-IR, Cary 630, Agilent.Santa Clara, CA, USA) was used to assess the functional groups of ion exchanger and possible metal binding mechanism after the La(III) and Ni(II) ions sorption. The prepared samples were placed on a diamond attachment. Each spectrum was recorded in the frequency range of 650–4000cm^−1^ with a spectral resolution of 4 cm^−1^ and a measurement time of 30 s. The baseline correction was performed using the Agilent MicroLab PC software (MicroLab FTIR software). The UHV multi-chamber analytical system (Prevac Ltd., Rogów, Poland) for X-ray photoelectron spectroscopy analyses was applied to evaluate the elemental composition on the samples surface before and after lanthanum(III) ions sorption. The structure and surface morphology of Lewatit Monoplus SP112 was analyzed by recording SEM images by the FEI Quanta 3D FEG scanning electron microscope. Prior to the measurements, the ion exchanger samples were sprayed with a gold layer using a high vacuum sprayer before and after La(III) loading. Microscopic images were taken for the samples using 100× and 5000× magnification. Moreover, the ion exchanger surface parameters such as surface area and pore texture were determined by the ASAP 2405 instrument (Micromeritics Inc.). Thermal stability of the ion exchanger was tested by thermogravimetry using Q50 TGA (TA Instruments, New Castle, DE, USA). Finally, 907 Titrando with the dosing units (Metrohm, Herisau, Switzerland) was applied to determine the pH of the point of zero charge (pH_pzc_) by means of the pH potentiometric titration method.

### 4.3. Batch and Column Experiments

The batch experiments were performed by the static method using the defined ion exchanger mass, HNO_3_ concentration, initial solution concentration, solution volume and stirring time. The ratio of ion exchanger dosage to the solution volume was 1:100. All experiments were performed in 100 cm^3^ Erlenmeyer flasks.

The effect of the HNO_3_ concentration was evaluated by weighing 0.1 g of ion exchanger to each flask and then 10 cm^3^ of 50 mg/dm^3^ La(III) or Ni(II) ions solution was transferred to the flasks. The HNO_3_ concentration was in the range 0.2–2 mol/dm^3^. The solutions were agitated in the shaker at 293 K for 360 min. After determination of the optimal HNO_3_ concentration, the effect of ion exchanger mass (0.1–0.5 g), phase contact time (1–360 min), initial metal concentration (25–200 mg/dm^3^) and temperature (293–333 K) was assessed. Kinetic studies were carried out with the varied initial La(III) or Ni(II) ions concentrations from 25 mg/dm^3^ to 200 mg/dm^3^. In addition, the isotherm studies were performed at different temperatures (293, 313, and 333 K) and different initial metal ions concentrations (25–1000 mg/dm^3^). After the sorption process the ion exchanger beads were separated and the liquid was analysed by the ICP-OES and FAAS to determine the La(III) and Ni(II) ions concentration in the liquid phase, respectively. The above experiments were conducted three times and the mean values were used in the data analysis. All analyses were made using the ORIGIN PRO8 software (OriginLab, Northampton, MA, USA).

The column experiments were performed using glass columns (diameter = 1 cm and height = 25 cm) packed with 10 cm^3^ of swollen ion exchanger. The initial La(III) and Ni(II) ions solution concentration was 50 mg/dm^3^. The pH of the prepared solutions was 1.5. Then La(III) or Ni(II) ions solution was passed through the prepared bed at a constant flow rate of 0.6 cm^3^/min. The column experiments were conducted at room temperature (293 K). After passing through the column the solution was collected into fractions and analysed by the ICP-OES or FAAS method. The column experiments were conducted until the La(III) and Ni(II) ions concentration in the effluent reached the initial metal concentration equal 50 mg/dm^3^. Regeneration of the ion exchanger was performed by passing 2 mol/dm^3^ HCl through the column bed at a constant flow rate of 0.6 cm^3^/min. The effluent solution was analysed for the La(III) and Ni(II) ions content.

The simultaneous sorption of metal ions from the binary systems was also performed to find out the effect of the presence of other metal ions, i.e., Ce(III), Pr(III), Nd(III), Fe(III), Ni(II), Co(II), Cu(II) and Zn(II) on the sorption efficiency of La(III) ions. The tests were carried out using the following parameters: m = 0.1 g, C_0_ = 50 mg/dm^3^, V = 10 cm^3^, CHNO_3_ = 0.2 mol/dm^3^, and t = 240 min, using the following systems: La-Ce, La-Pr, La-Nd, La-Fe, La-Ni, La-Co, La-Cu and La-Zn.

### 4.4. Calculations

#### 4.4.1. Basic Parameters of the Batch Experiments

The amount metal ions adsorbed on the ion exchanger at the defined time t(*q_t_*) [mg/g] and the equilibrium (*q_e_*) [mg/g] as well as the sorption *%S* and desorption *%D* percentage, and the distribution coefficient (*K_d_*) [cm^3^/g] were calculated from the following equations:

(3) $$q_{t}=\left(C_{0}-C_{t}\right)\times \frac{V}{m}$$

(4) $$q_{e}=\left(C_{0}-C_{e}\right)\times \frac{V}{m}$$

(5) $$\%S=\frac{C_{0}-C_{t}}{C_{0}}\times 100\%$$

(6) $$\%D=\frac{C_{des}}{C_{0}}\times 100\%$$

(7) $$K_{d}=\frac{\left(C_{0}-C_{t}\right)}{C_{t}}\times \frac{V}{m}$$

where *C_0_* is the initial La(III) and Ni(II) ions concentration [mg/dm^3^], *C_t_* is the La(III) and Ni(II) ions concentration after time t [mg/dm^3^], *V* is the solution volume [dm^3^], *m* is the ion exchanger dosage [g], and *C_des_* is the La(III) and Ni(II) ions concentration after the desorption process [mg/dm^3^].

#### 4.4.2. Basic Parameters of the Dynamic Experiments

Based on the breakthrough curves (*C*/*C_0_* as a function of *V*), the adsorption capacities (*q_ec_*) [mg/g], the total (*C_t_*) and working (*C_w_*) exchange capacities [mg/cm^3^], and also the volumetric (*D_v_*) and mass (*D_g_*) distribution coefficients were estimated:

(8) $$q_{ec}=\frac{U\times C_{0}\times C_{ec}}{m_{j}}$$

(9) $$C_{t}=\frac{\overset{-}{U}\times C_{0}}{V_{j}}$$

(10) $$C_{w}=\frac{U\times C_{0}}{V_{j}}$$

(11) $$D_{v}=\frac{\overset{-}{U}-U_{0}-V}{V_{j}}$$

(12) $$D_{g}=\frac{\overset{-}{U}-U_{0}-V}{m_{j}}$$

where: *C_0_* and *C_ec_* is the initial and equilibrium (at breakthrough) metal ions concentration [mg/dm^3^], *U* is the total leakage volume until the breakthrough point [cm^3^], *m_j_* is the dry bed mass [g], the $\overset{-}{U}$ is the volume of the leakage equal *C*/*C_0_* = 0.5 [cm^3^], *V_j_* is the resin bed volume placed in the glass column [cm^3^], *U_0_* is the volume of dead column [cm^3^] and *V* is the empty space volume between the resin beads equal 0.4 cm^3^.

#### 4.4.3. Kinetic Parameters

The experimental kinetic parameters for the studied metal ions were calculated using the following equations:

a) the Lagergren pseudo-first order (PFO):
  (13) $$\log(q_{1}-q_{t})=\log(q_{1})-\frac{k_{1}}{2.303}\times t$$

b) the Ho-McKay pseudo-second order (PSO) [27]:
  (14) $$\frac{t}{q_{t}}=\frac{1}{k_{2}\times q_{2}^{2}}+\frac{1}{q_{2}}\times t$$

c) the intraparticle Weber-Morris diffusion (IPD):
  (15) $$q_{t}=k_{i}\times t^{1/2}+C_{i}$$

d) the Boyd model:
  (16) $$\frac{q_{t}}{q_{e}}=1-\frac{6}{\pi^{2}}\exp(-Bt)$$
  (17) $$Bt=0.4977-\ln(1-\frac{q_{t}}{q_{e}})$$

e) the film diffusion model:
  (18) $$k_{f}t=\ln(1-F)$$

f) the pore diffusion coefficient (*D_p_*) [cm^2^/s]:
  (19) $$D_{p}=\frac{0.03r^{2}}{t_{1/2}}$$

g) the film diffusion coefficient (*D_f_*) [cm^2^/s]:
  (20) $$D_{f}=\frac{0.23r\delta q_{e}}{t_{1/2}C_{0}}$$

where: *k_1_* is the PFO rate constant [1/min], *q_1_* is the equilibrium capacity according to the PFO model [mg/g], *k_2_* is the PSO rate constant [g/mg·min], *q_2_* is the sorption equilibrium capacity according to the PSO model [mg/g], *k_i_* is the IPD rate constants [mg/g·min^1/2^], *C_i_* indicates the boundary layer thickness, *Bt* is the *q_t_*/*q_e_* function, *k_f_* is the rate constant of film diffusion [1/min], *F* is the constant equal *q_t_/q_e_*, *r* is the resin radius [cm], *t_1/2_* is the time at which the maximum sorption was reached [s], and *δ* is the film thickness (equal 1 × 10^−3^ cm).

#### 4.4.4. Isotherm Parameters

The adsorption equilibrium parameters of La(III) and Ni(II) ions adsorption onto Lewatit Monoplus SP112 were determined using the following equations:

a) the Langmuir isotherm model:
  (21) $$\frac{C_{e}}{q_{e}}=\frac{1}{K_{L}q_{m}}+\frac{C_{e}}{q_{m}}$$

b) the Freundlich isotherm model:
  (22) $$\log q_{e}=\log K_{F}+\frac{1}{n}\log C_{e}$$

c) the Temkin isotherm model:
  (23) $$q_{e}=B\ln A+B\ln C_{e}$$

where: *K_L_* is the Langmuir constant [dm^3^/mg], *q_m_* is the maximum adsorption capacity [mg/g], *K_F_* [mg/g] and 1/*n* are the Freundlich constants and 1/*n* indicates the adsorption favourability, *B* is the Temkin constant [J/mol] and can be written as *B* = *RT*/*b_T_*, where *b_T_* is the constant associated with the sorption heat [kJ/mol], and *A* is the Temkin constant corresponding to the maximum binding energy [dm^3^/g].

Additionally, two errors functions, i.e., the Chi-square (*Χ^2^*) and root mean square error (*RMSE*) were used for isotherm models fitting:

(24) $$\chi^{2}=\sum \frac{{\left(q_{e,\exp}-q_{e,cal}\right)}^{2}}{q_{e,cal}}$$

(25) $$RMSE=\sqrt{\frac{1}{n-1}\sum_{n=1}^{n}{\left(q_{e,\exp}-q_{e,cal}\right)}^{2}}$$

where: *q_e,exp_* is the adsorption capacity determined experimentally, *q_e,cal_* is the adsorption capacity according to the given isotherm model, and *n* is the numer of repetitions.

#### 4.4.5. Thermodynamic Parameters

The thermodynamic parameters of the La(III) and Ni(II) ions sorption onto Lewatit Monoplus SP112, i.e., the Gibbs free energy (*ΔG°*), the enthalpy (*ΔH°*) and entropy (*ΔS°*) change were determined from the following equations:

(26) $$\Delta G{}^{\circ}=-RT\ln K_{c}$$

(27) $$K_{c}=\frac{q_{e}}{C_{e}}$$

(28) $$\ln K_{c}=\frac{\Delta S{}^{\circ}}{R}-\frac{\Delta H{}^{\circ}}{RT}$$

where: *K_c_* is the distribution constant at equilibrium [dm^3^/g].

## Figures and Tables

**Table 1:** Characteristics of Lewatit Monoplus SP112.

Characteristics	Value
Matrix	Crosslinked polystyrene and divinylbenzene
Physical form	Spherical beige-gray beads
Functional groups	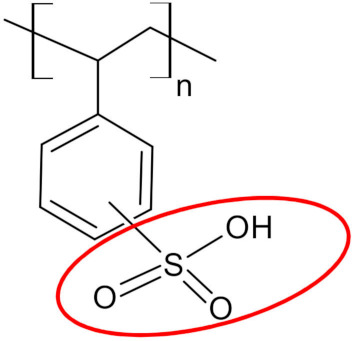 sulfonic acid
Ionic form	Na^+^
Total exchange capacity [val/dm^3^]	1.7
Average grain size [mm]	0.65
Bulk density [g/dm^3^]	740
Uniformity coefficient	1.10
pH operating range	0–14
Maximum temperature operating [K]	393
pH_pzc_	6.61
S_BET_ [m^2^/g]	14.98
D [nm]	32.72
V [cm^3^/g]	0.144
Image	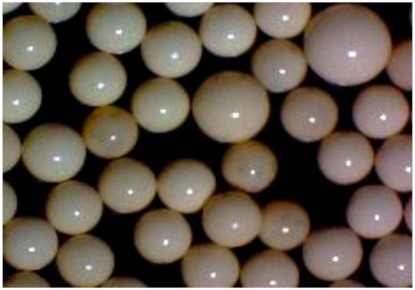

**Table 2:** Kinetic parameters for the La(III) and Ni(II) ions sorption on Lewatit Monoplus SP112.

**C_0_** **[mg/dm^3^]**	**q_exp_** **[mg/g]**		**Pseudo-First Order**					**Pseudo-Second Order**			
			**q_1_** **[mg/g]**	**k_1_** **[1/min]**			**R^2^**	**q_2_** **[mg/g]**		**k_2_** **[g/mg·min]**	**R^2^**
La(III)											
25	3.13		0.47		0.055		0.949	3.14		0.496	0.999
50	6.44		0.59		0.044		0.711	6.46		0.171	0.999
100	12.41		1.00		0.049		0.769	12.45		0.121	0.999
150	17.15		1.64		0.041		0.724	17.21		0.071	0.999
200	23.24		2.36		0.046		0.803	23.31		0.054	0.999
Ni(II)											
25	2.35		0.36		0.023		0.679	2.36		0.346	1.000
50	4.31		0.79		0.030		0.839	4.33		0.185	1.000
100	9.27		1.44		0.026		0.756	9.30		0.097	1.000
150	12.64		1.84		0.018		0.579	12.66		0.063	1.000
200	16.56		3.33		0.018		0.662	16.61		0.032	0.999
**C_0_** **[mg/dm^3^]**	**Weber-Morris Intraparticle Diffusion**									**Boyd**	
	k_i1_^*^	**C_1_**	**R^2^**	k_i2_^*^	**C_2_**	**R^2^**	k_i3_^*^	**C_3_**	**R^2^**	**Bt**	**R^2^**
La(III)											
25	0.86	0.74	0.977	0.03	2.91	0.608	0.01	3.09	0.509	0.003	0.949
50	2.31	0.79	0.984	0.10	5.73	0.657	0.01	6.43	0.954	0.005	0.711
100	3.95	1.16	0.978	0.15	11.40	0.656	0.01	12.40	0.832	0.005	0.769
150	5.62	1.44	0.976	0.27	15.31	0.645	0.01	17.12	0.845	0.005	0.724
200	7.88	1.46	0.965	0.34	20.89	0.598	0.02	23.21	0.746	0.005	0.803
Ni(II)											
25	0.76	0.08	0.993	0.04	2.02	0.807	0.01	2.30	0.986	0.003	0.679
50	1.45	0.13	0.989	0.08	3.67	0.848	0.01	4.23	0.816	0.003	0.839
100	2.93	0.74	0.991	0.17	7.98	0.850	0.01	9.09	0.891	0.003	0.757
150	3.46	2.06	0.970	0.24	10.77	0.622	0.02	12.25	0.958	0.003	0.579
200	4.17	2.45	0.950	0.33	13.78	0.724	0.05	15.70	0.952	0.002	0.663
**C_0_** **[mg/dm^3^]**	**Film Diffusion Model**					**Film Diffusion Coefficient** **D_f_ [cm^2^/s]**			**Pore Diffusion Coefficient** **D_p_ [cm^2^/s]**		
	**k_f_ [1/min]**			**R^2^**							
La(III)											
25	11.43			0.949		2.04 × 10^−7^			1.76 × 10^−8^		
50	9.54			0.711		1.72 × 10^−6^			1.76 × 10^−8^		
100	9.38			0.769		6.12 × 10^−6^			1.76 × 10^−8^		
150	8.77			0.724		6.40 × 10^−6^			8.80 × 10^−9^		
200	8.73			0.803		9.12 × 10^−6^			8.80 × 10^−9^		
Ni(II)											
25	6.58			0.680		1.29 × 10^−7^			8.80 × 10^−9^		
50	6.02			0.840		4.56 × 10^−7^			8.80 × 10^−9^		
100	5.26			0.757		2.04 × 10^−6^			8.80 × 10^−9^		
150	4.42			0.590		2.59 × 10^−6^			5.97 × 10^−9^		
200	4.37			0.663		6.76 × 10^−6^			5.97 × 10^−9^		

k_i1_*, k_i2_*, k_i3_* [mg/g·min^1/2^].

**Table 3:** Isotherm parameters for the La(III) and Ni(II) ions sorption on LewatitMonoplus SP112.

**T** **[K]**	**q_exp_** **[mg/g]**	**Langmuir**								
		***q_m_*** **[mg/g]**		**K_L_** **[dm^3^/mg]**		**R^2^**		**Χ^2^**		**RMSE**
La(III)										
293	92.06	93.21		1.169		0.999		0.006		0.56
313	92.90	94.61		1.192		0.996		0.009		0.67
333	93.74	95.34		1.702		0.998		0.014		0.81
Ni(II)										
293	48.64	55.30		0.012		0.990		0.344		0.21
313	49.60	59.17		0.018		0.991		0.073		1.33
333	53.32	60.81		0.019		0.998		0.004		0.32
**T** **[K]**	**Freundlich**					**Temkin**				
	***K_F_*** **[mg/g]**	**N**	**R^2^**	**Χ^2^**	**RMSE**	***A*** **[dm^3^/g]**	***B*** **[J/mol]**	**R^2^**	**Χ^2^**	**RMSE**
La(III)										
293	35.42	2.11	0.688	161.91	127.63	27.093	163.82	0.921	1.66	9.32
313	37.73	2.22	0.594	104.55	95.99	31.621	161.02	0.904	1.54	8.99
333	44.44	2.08	0.699	150.14	121.91	43.038	163.41	0.896	0.98	7.15
Ni(II)										
293	1.380	1.53	0.959	21.01	27.22	0.217	246.91	0.936	1.03	4.79
313	2.227	1.74	0.957	15.37	22.90	0.368	273.27	0.959	1.38	5.56
333	2.251	1.64	0.954	26.37	32.44	0.372	242.89	0.953	0.81	4.50

**Table 4:** Comparison of La(III) and Ni(II) ions sorption capacities by other materials.

Sorbents	Conditions			q_e_ [mg/g]	Literature
	pH	t [min]	T [K]		
La(III) ions sorption					
Kaolinite	5.0	360	323	2.75	[41]
Magnetic silica nanocomposite	5.5	30	298	55.90	[46]
Pectin from durian rind	4.0	90	298	41.20	[47]
Poly-γ-glutamic acid crosslinked with polyvinyl alcohol	6.0	60	303	8.99	[48]
Lewatit Monoplus SP112	1.5	30	333	93.74	This study
Ni(II) ions sorption					
Lewatit Monoplus SP112Amberlite 200CAmberlyst 15	1.0 1.0 1.0	30 30 30	- - -	33.73 34.20 29.57	[18]
Natural clay	5.5	120	298	6.25	[49]
Silica-based hybrid adsorbent	-	10 080	298	49.24	[50]
LewatitMonoplus SP112	1.5	60	333	53.32	This study

“-”no information about a parameter value.

**Table 5:** Thermodynamic parameters for the La(III) and Ni(II) ions sorption on Lewatit Monoplus SP112.

T [K]	K_c_ [dm^3^/g]	ΔH° [kJ/mol]	ΔS° [J/mol·K]	ΔG° [kJ/mol]
La(III)				
293	58.34			−26.73
313	75.96	15.41	76.40	−29.24
333	108.59			−32.10
Ni(II)				
293	0.55			−15.38
313	1.09	12.54	48.40	−18.21
333	1.17			−19.55

**Table 6:** Dynamic studies parameters of lanthanum(III) and nickel(II) ions sorption (C_0_ = 50 mg/dm^3^, pH = 1.5).

U [cm^3^]	$\overset{-}{U}$ [cm^3^]	q_ec_ [mg/g]	C_t_ [mg/cm^3^]	C_w_ [mg/cm^3^]	D_g_	D_v_	%D
La(III)							
9200	12050	49.12	53.77	41.05	1442.59	1204.56	99.96
Ni(II)							
1800	2550	10.86	12.85	9.07	304.86	254.56	99.94

## References

[1] Sun Z., Xiao Y., Sietsma J., Agterhuis H., Yang Y. (2015). A cleaner process for selective recovery of valuable metals from electronic waste of complex mixtures of end-of-life electronic products. Environ. Sci. Technol..

[2] Kaya M. (2016). Recovery of metals and nonmetals from electronic waste by physical and chemical recycling processes. Waste Manag..

[3] Abdelbasir S.M., Hassan S.S.M., Kamel A.H., El-Nasr R.S. (2018). Status of electronic waste recycling techniques: A review. Environ. Sci. Pollut. Res..

[4] Balde C.P., Forti V., Gray V., Kuehr R., Stegmann P. (2017). The Global E-Waste Monitor-2017.

[5] Zhang S., Huang X., Wang D. (2015). Review on comprehensive recovery of valuable metals from spent electrode materials of nickel-hydrogen batteries. Rare Met. Mater. Eng..

[6] Nan J., Han D., Yang M., Cui M., Hou X. (2006). Recovery of metal values from a mixture of spent lithium-ion batteries and nickel-metal hydride batteries. Hydrometallurgy.

[7] Ordoñez J., Gago E.J., Girard A. (2016). Processes and technologies for the recycling and recovery of spent lithium-ion batteries. Renew. Sustain. Energy Rev..

[8] Dominish E., Teske S., Florin N. (2019). Responsible minerals sourcing for renewable energy. Report Prepared for Earthworks by the Institute for Sustainable Futures.

[9] Sun X., Li Z., Wang X., Li C. (2020). Technology development of electric vehicles: A review. Energies.

[10] Al-Thyabat S., Nakamura T., Shibata E., Iizuka A. (2013). Adaptation of minerals processing operations for lithium-ion (LiBs) and nickel metal hydride (NiMH) batteries recycling: Critical review. Miner. Eng..

[11] Lin S.L., Huang K.L., Wang I.C., Chou I.C., Kuo Y.M., Hung C.H., Lin C. (2016). Characterization of spent nickel–metal hydride batteries and a preliminary economic evaluation of the recovery processes. J. Air Waste Manag. Assoc..

[12] Porvali A., Ojanen S., Wilson B.P., Serna-Guerrero R., Lundström M. (2020). Nickel metal hydride battery waste: Mechano-hydrometallurgical experimental study on recycling aspects. J. Sustain. Metall..

[13] Agarwal V., Khalid M.K., Porvali A., Wilson B.P., Lundström M. (2019). Recycling of spent NiMH batteries: Integration of battery leach solution into primary Ni production using solvent extraction. Sustain. Mater. Technol..

[14] Savov G., Angelov T., Tsekov A.L., Grigorova I., Nishkov I. Combination of ion exchange and solvent extraction versus solvent extraction: A technical-economical comparison. Proceedings of the 26th International Mineral Processing Congress, IMPC 2012: Innovative Processing for Sustainable Growth-Conference Proceedings.

[15] Bao S., Tang Y., Zhang Y., Liang L. (2016). Recovery and Separation of Metal Ions from Aqueous Solutions by Solvent-Impregnated Resins. Chem. Eng. Technol..

[16] Makanyire T., Sanchez-Segado S., Jha A. (2016). Separation and recovery of critical metal ions using ionic liquids. Adv. Manuf..

[17] Mohebbi A., Mahani Abolghasemi A., Izadi A., Inamuddin Rangreez T.A., Asiri A.M. (2019). Ion exchange resin technology in recovery of precious and noble metals. Applications of Ion Exchange Materials in Chemical and Food Industries.

[18] Otrembska P., Gega J. (2013). Kinetic studies on sorption of Ni(II) and Cd(II) from chloride solutions using selected acidic cation exchangers. Physicochem. Probl. Miner. Process..

[19] Lin L.C., Li J.K., Juang R.S. (2008). Removal of Cu(II) and Ni(II) from aqueous solutions using batch and fixed-bed ion exchange processes. Desalination.

[20] Koopman C., Witkamp G.J. (2000). Extraction of lanthanides from the phosphoric acid production process to gain a purified gypsum and a valuable lanthanide by-product. Hydrometallurgy.

[21] Khawassek Y.M., Eliwa A.A., Haggag E.S.A., Omar S.A., Abdel-Wahab S.M. (2019). Adsorption of rare earth elements by strong acid cation exchange resin thermodynamics, characteristics and kinetics. SN Appl. Sci..

[22] Rychkov V.N., Kirillov E.V., Kirillov S.V., Bunkov G.M., Mashkovtsev M.A., Botalov M.S., Semenishchev V.S., Volkovich V.A. (2016). Selective ion exchange recovery of rare earth elements from uranium mining solutions. Proceedings of the AIP Conference Proceedings.

[23] Lokshin E.P., Tareeva O.A., Elizarova I.R. (2015). Sorption of rare-earth elements from phosphogypsum sulfuric acid leaching solutions. Theor. Found. Chem. Eng..

[24] Xu J., Virolainen S., Zhang W., Kuva J., Sainio T., Koivula R. (2018). Polyacrylonitrile-encapsulated amorphous zirconium phosphate composite adsorbent for Co, Nd and Dy separations. Chem. Eng. J..

[25] Virolainen S., Repo E., Sainio T. (2019). Recovering rare earth elements from phosphogypsum using a resin-in-leach process: Selection of resin, leaching agent, and eluent. Hydrometallurgy.

[26] Kołodyńska D., Fila D., Hubicki Z. (2020). Evaluation of possible use of the macroporous ion exchanger in the adsorption process of rare earth elements and heavy metal ions from spent batteries solutions. Chem. Eng. Process. Process Intensif..

[27] Socrates G. (2001). Infrared and Raman characteristic group frequencies. Tables and Charts.

[28] Lei Y., Cui Y., Huang Q., Dou J., Gan D., Deng F., Liu M., Li X., Zhang X., Wei Y. (2019). Facile preparation of sulfonic groups functionalized Mxenes for efficient removal of methylene blue. Ceram. Int..

[29] Brijmohan S.B., Swier S., Weiss R.A., Shaw M.T. (2005). Synthesis and characterization of cross-linked sulfonated polystyrene nanoparticles. Ind. Eng. Chem. Res..

[30] Dizge N., Keskinler B., Barlas H. (2009). Sorption of Ni(II) ions from aqueous solution by Lewatit cation-exchange resin. J. Hazard. Mater..

[31] Edebali S., Pehlivan E. (2016). Evaluation of chelate and cation exchange resins to remove copper ions. Powder Technol..

[32] Guo H., Ren Y., Sun X., Xu Y., Li X., Zhang T., Kang J., Liu D. (2013). Removal of Pb^2+^ from aqueous solutions by a high-efficiency resin. Appl. Surf. Sci..

[33] Siow K.S., Britcher L., Kumar S., Griesser H.J. (2018). XPS study of sulfur and phosphorus compounds with different oxidation states. Sains Malaysiana.

[34] Meng Q., Cai Y., Cong B., Xing W., Chen G. (2020). Enhanced carriers separation efficiency in g-C_3_N_4_ modified with sulfonic groups for efficient photocatalytic Cr(VI) reduction. Mater. Res. Bull..

[35] Sarbak Z. (2000). Adsorption and Adsorbents: Theory and Application.

[36] Ling P., Liu F., Li L., Jing X., Yin B., Chen K., Li A. (2010). Adsorption of divalent heavy metal ions onto IDA-chelating resins: Simulation of physicochemical structures and elucidation of interaction mechanisms. Talanta.

[37] Kawamura F., Matsuda M., Aoyama Y., Chino K., Mizumoto M. (1987). Method of Disposing Radioactive Ion Exchange Resin. U.S. Patent.

[38] Ho Y.S., McKay G. (1999). Pseudo-second order model for sorption processes. Process Biochem..

[39] Kumar R., Ansari M.O., Alshahrie A., Darwesh R., Parveen N., Yadav S.K., Barakat M.A., Cho M.H. (2019). Adsorption modeling and mechanistic insight of hazardous chromium on para toluene sulfonic acid immobilized-polyaniline@CNTs nanocomposites. J. Saudi Chem. Soc..

[40] Michelsen D.L., Gideon J.A., Griffith G.P., Pace J.E., Kutat H.L. (1975). Removal of soluble mercury from waste water by complexing techniques. Virginia Water Resources Research Center Bulletin 74.

[41] Zhou F., Feng J., Xie X., Wu B., Liu Q., Wu X., Chi R. (2019). Adsorption of lanthanum(III) and yttrium(III) on kaolinite: Kinetics and adsorption isotherms. Physicochem. Probl. Miner. Process..

[42] Kiruba U.P., Kumar P.S., Prabhakaran C., Aditya V. (2014). Characteristics of thermodynamic, isotherm, kinetic, mechanism and design equations for the analysis of adsorption in Cd(II) ions-surface modified *Eucalyptus seeds* system. J. Taiwan Inst. Chem. Eng..

[43] Hamzaoui M., Bestani B., Benderdouche N. (2018). The use of linear and nonlinear methods for adsorption isotherm optimization of basic green 4-dye onto sawdust-based activated carbon. J. Mater. Environ. Sci..

[44] Vandenbossche M., Jimenez M., Casetta M., Traisnel M. (2015). Remediation of heavy metals by biomolecules: A review. Crit. Rev. Environ. Sci. Technol..

[45] Côté A.P., Shimizu G.K.H. (2003). The supramolecular chemistry of the sulfonate group in extended solids. Coord. Chem. Rev..

[46] Wu D., Sun Y., Wang Q. (2013). Adsorption of lanthanum (III) from aqueous solution using 2-ethylhexyl phosphonic acid mono-2-ethylhexyl ester-grafted magnetic silica nanocomposites. J. Hazard. Mater..

[47] Kusrini E., Wicaksono W., Gunawan C., Daud N.Z.A., Usman A. (2018). Kinetics, mechanism, and thermodynamics of lanthanum adsorption on pectin extracted from durian rind. J. Environ. Chem. Eng..

[48] Gao Y., Zhang S., Zhao K., Wang Z., Xu S., Liang Z., Wu K. (2015). Adsorption of La^3+^ and Ce^3+^ by poly-γ-glutamic acid crosslinked with polyvinyl alcohol. J. Rare Earths.

[49] Es-Sahbany H., Berradi M., Nkhili S., Hsissou R., Allaoui M., Loutfi M., Bassir D., Belfaquir M., El Youbi M.S. (2019). Removal of heavy metals (nickel) contained in wastewater-models by the adsorption technique on natural clay. Mater. Today Proc..

[50] Xu M., Liu J., Hu K., Xu C., Fang Y. (2016). Nickel(II) removal from water using silica-based hybrid adsorbents: Fabrication and adsorption kinetics. Chin. J. Chem. Eng..

[51] Srivastava V.C., Mall I.D., Mishra I.M. (2006). Modelling individual and competitive adsorption of cadmium(II) and zinc(II) metal ions from aqueous solution onto bagasse fly ash. Sep. Sci. Technol..

[52] Girish C.R. (2018). Multicomponent adsorption and the interaction between the adsorbent and the adsorbate: A review. Int. J. Mech. Eng. Technol..

[53] Dong C., Zhang F., Pang Z., Yang G. (2016). Efficient and selective adsorption of multi-metal ions using sulfonated cellulose as adsorbent. Carbohydr. Polym..

